# Characterization and Gene Mapping of an Open-Glume *Oryza sativa* L. Mutant

**DOI:** 10.3390/ijms241612702

**Published:** 2023-08-11

**Authors:** Xingxue Mao, Xiaoyu Zheng, Wenfeng Chen, Chen Li

**Affiliations:** 1 Rice Research Institute, Guangdong Academy of Agricultural Sciences, Guangzhou 510640, China; 2 Guangdong Key Laboratory of New Technology in Rice Breeding, Guangzhou 510640, China

**Keywords:** rice, BSA, floral organs development, *OsJAG*

## Abstract

Floral organ development determines agricultural productivity by affecting seed development, seed quality, and final yield. In this study, we described the novel *ogl* mutant in rice (*Oryza sativa* L.), which is characterized by an open-glume phenotype, increased pistil number, reduced stamen number, decreased seed setting rate, and smaller rice grains. Genetic analysis showed that the open-glume phenotype might be controlled by a recessive qualitative trait locus. Employing bulked segregant analysis (BSA), one candidate region was identified on rice chromosome 1. The glume opening phenotype cosegregated with SNP (Chr1:1522703), which was located at the start codon of one transcript of *OsJAG*, resulting in partial loss of *OsJAG* function. cDNA analysis revealed that *OsJAG* encodes two transcript variants. Compared to normal plants, the expression of *OsJAG.1* was upregulated in open-glume plants. When investigating the glume phenotype, we found that the expression of genes related to floral development changed greatly in open-glume plants. Taken together, this work increases our understanding of the developmental role of *OsJAG* in rice floral development.

## 1. Introduction

Rice (*Oryza sativa* L.) is one of the major food crops in the world, serving as a caloric foundation for more than half of the world’s population [1,2,3]. Seed yield is the ultimate goal of rice production, which is dependent on floral organ development. Numerous genes related to flower development have been demonstrated to regulate seed development and final yield [4,5,6]. Even the development of glumes influences seed yield [7,8]. In hybrid rice, glume opening is beneficial for pollen dispersal and pollen acceptance on the pistil, which is an indispensable and favorable reproductive trait [9,10,11]. Usually, the glume determines grain size and protects the developing seeds during the filling stage and the mature stages, especially in high-temperature and humidity conditions [12,13]. Glume opening renders rice susceptible to microbial invasion and rainwater infiltration, which can result in seed deterioration and preharvest sprouting. Severe glume opening often leads to inactive seeds. In a word, every floral organ is closely related to seed development [5,14,15], so understanding floral organ development is of great significance to inform agricultural practices.

Numerous genes have been reported to influence the development of flower organs in rice. For example, the MADS (minichromosome maintenance 1, AGAMOUS, DEFICIENS, Serum response factor) family of transcription factors prominently controls floret development [15,16,17]. *OsMADS1* mutants exhibit phenotypic variation among different alleles, including leaf-like glumes, naked rice, glume-like lodicules, altered morphology, and the numbers of stamens and pistils [18,19,20,21,22]. Mutation of *OsMADS15* causes shrunken paleae and uncoordinated development of paleae and lemma that result in incomplete closure of florets [23]. Interestingly, double mutations in *OsMADS1* and *OsMADS15* result in asexual reproduction in rice [23]. A well-characterized heterosis gene, *GW3p6*, which is an allele of *OsMADS1* with an altered splicing pattern that truncates 32 amino acid residues at the C-terminus, causes an increase in grain length, grain weight, and yield [24]. OsMADS1 interacts with GS3, DEP1, and GGC2 to regulate rice-grain shape [6,25]. *WG7* can also affect grain width by regulating *OsMADS1* transcription [26].

Aside from MADS genes, other rice mutants from various genes have been implicated in glume formation and integrity. *BSG1* encodes a DUF640 domain protein of unknown function and a *bsg1* mutant has shown loosely interlocked lemmas and paleae, beak-shaped grains, and an approximate 50% decrease in 1000-grain weight [4]. OsWOX3A encodes a transcriptional activator that causes unclosed florets and fewer spikelets per panicle when mutated [27]. Overexpression of microRNA172 family genes leads to glume opening, longer lemma and palea, and abnormal floral organ development [14]. *OsJAG* (*SL1*) encodes a C2H2 zinc finger transcription factor. Mutants in *OsJAG* display open-glume phenotypes, distorted paleae/lemmas, greater numbers of pistils, fewer or missing stamen, and male-sterile florets [28,29]. Arabidopsis mutants in *AtJAG* (*jag-1*) have stunted sepals and anthers than their wild-type counterparts with male-sterile flowers [30].

In this study, we characterized a recessive mutant that we refer to as open-glume rice (*ogl*). Via BSA, we discovered that *ogl* harbors a novel allele of the *OsJAG* gene (*OsJAG^ogl^*), which has a point mutation that causes the start-loss of one transcript. Further analysis showed that the point mutation in *OsJAG^ogl^* is associated with an open-glume phenotype. Furthermore, the expression levels of genes related to floret development were altered in open-glume plants. Taken together, this work bolsters our understanding of the role of *OsJAG* in rice floral organ development and glume formation.

## 2. Results

### 2.1. Characterization of the ogl Rice Mutant

**Figure 1:**
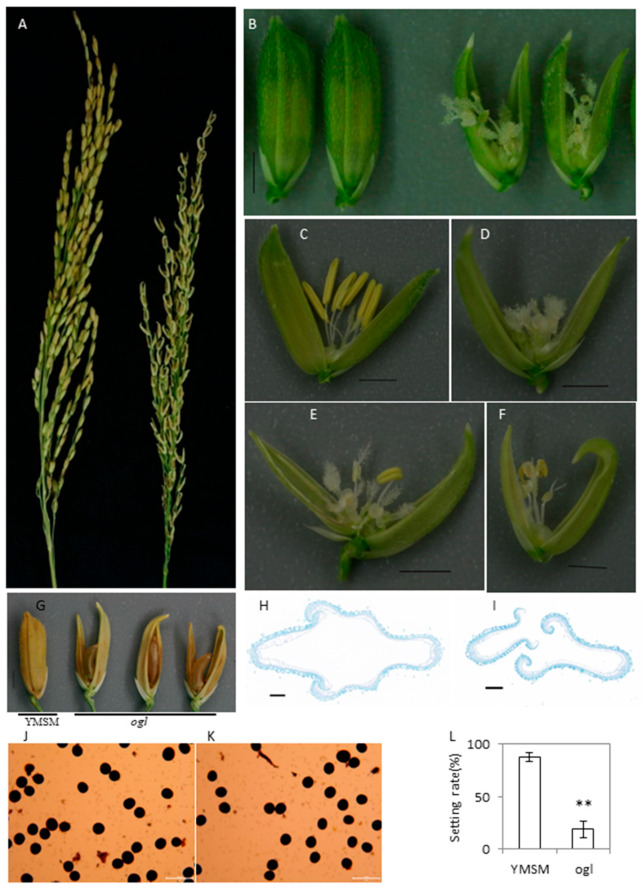
Morphological characteristics of the *ogl* mutant. (**A**) Panicle morphology of YMSM (left) and *ogl* (right). (**B**) The floret of YMSM (left) and *ogl* (right), bar = 2 mm. (**C**–**F**) The floret of YMSM (**C**) and *ogl* (**D**–**F**) after opening the glumes, bar = 2 mm. (**G**) The mature grains of YMSM and *ogl*, bar = 2 mm. (**H**,**I**) Transverse section from the middle of glume of YMSM (**H**) and *ogl* (**I**), bar = 200 μm. (**J**,**K**) I_2_-KI staining results of pollen grains of YMSM (**J**) and *ogl* (**K**), bar = 100 μm. (**L**) Comparison of setting rate between YMSM and *ogl.* Student’s t-tests were used to generate *p*-values. (** *p* < 0.01).

The *ogl* mutant is a spontaneous mutant that was discovered in a paddy field growing diverse indica rice varieties. As such, we deduced that *ogl* is of indica background. There was no significant difference in the vegetative tissues of *ogl* and YMSM (Yuanmeisimiao), an *indica* rice cultivar showing normal florets. The *ogl* mutant was originally discovered for its recognizable open-glume phenotype (Figure 1A,B,G). A normal rice floret is composed of one pistil (two stigmas) and six stamens (Figure 1C). In the *ogl* mutant, there are 0–6 stamens and multiple pistils in the floret (Figure 1D–F). One week after anthesis, YMSM florets remained in a glume-closed state but were open in *ogl* florets (Figure 1A). Before heading, the florets were collected and transversely sectioned across the middle to observe the glume. In YMSM, the lemma curled inward, the palea curled outward, and both clasped each other at the edge (Figure 1H). In *ogl*, the lemma and palea were similar in appearance to YMSM but they did not clasp each other (Figure 1I), suggesting that unsynchronized development might be the main reason for the abnormal glume. Although the development of stamens was abnormal and the number of anthers decreased, I_2_-KI staining revealed normal pollen development (Figure 1J,K). The self-seed setting rate of *ogl* decreased significantly (Figure 1L), which also indicated that the successfully developed anthers contained active pollen despite a decrease in the number of anthers.

### 2.2. Genetic Analysis of the Open-Glume Phenotype

The open-glume phenotype of *ogl-inbred* lines was stable in three consecutive generations, which indicates that the phenotype was heritable. To identify the gene responsible for the open-glume phenotype, we constructed a mapping population derived from a cross between *ogl* and YMSM. All of the F_1_ plants showed a normal floret phenotype, suggesting that the open-glume phenotype was a recessive trait. In the F_2_ populations, the segregation ratios of normal to open-glumes were more than 6.0 (Table 1), which deviated from a typical ratio of 3:1. We speculated that the low ratio of open-glume plants might be caused by anther and reproductive defects and the open-glume phenotype might be controlled by one recessive gene.

### 2.3. Cloning of the OsJAG^ogl^ Gene

**Figure 2:**
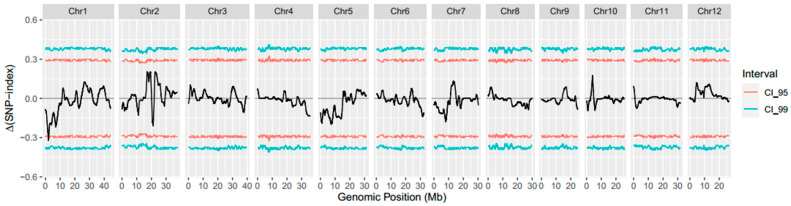
Distribution of △(SNP index) between two extreme pools. The black line represents △(SNP index). The red lines represent thresholds of 95%. The cyan lines represent thresholds of 99%.

To identify the open-glume-conferring gene, the extreme phenotype pools were constructed using the F_2_ population for bulked segregation analysis (BSA). A total of 59.9 Gb of effective data were obtained from the sequencing results. The sequencing data from the normal floret and glume-open floret pools were 26.9 Gb and 22.3 Gb, respectively. The Q30 of each pool was greater than 93.68%. With R498 as the reference genome (www.mbkbase.org/R498, accessed on 10 January 2023), 2,868,510 SNPs were identified in the normal floret pool and 2,885,176 SNPs in the open-glume pool. BSA-Seq data analysis was performed with TBtools [31]. For thresholding, 95% confidence levels were selected and a candidate region (14,759~7,736,351 bp) was identified on the short arm of chromosome 1 (Figure 2). This result supported the deduction that the open-glume phenotype was controlled by a recessive gene. First, we screened 358 exonic SNPs of 157 genes in the candidate region. Among them, 7 SNPs resulted in aberrant start codons or stop codons (Table 2). Based on the SNP data of MBKBASE (www.mbkbase.org, accessed on 10 March 2023), SNP (Chr1: 1522703) was not previously reported, suggesting that it was a novel variant.

According to the annotation information, SNP (Chr1: 1522703) is located within gene OsR498G0100098300.01, which encodes a single C_2_H_2_ zinc finger protein, *OsJAG*, known to regulate the development of floral organs in rice [28,29]. We designed primers to check the mutation in *OsJAG*. The DNA of YMSM, *ogl*, and F_1_ plants were used as templates for amplification. The sequencing results showed that the base transversion SNP (Chr1:1522703) occurred in *OsJAG* in the *ogl* genome and resulted in a new *OsJAG* allele, which was then designated as *OsJAG^ogl^.*

**Figure 3:**
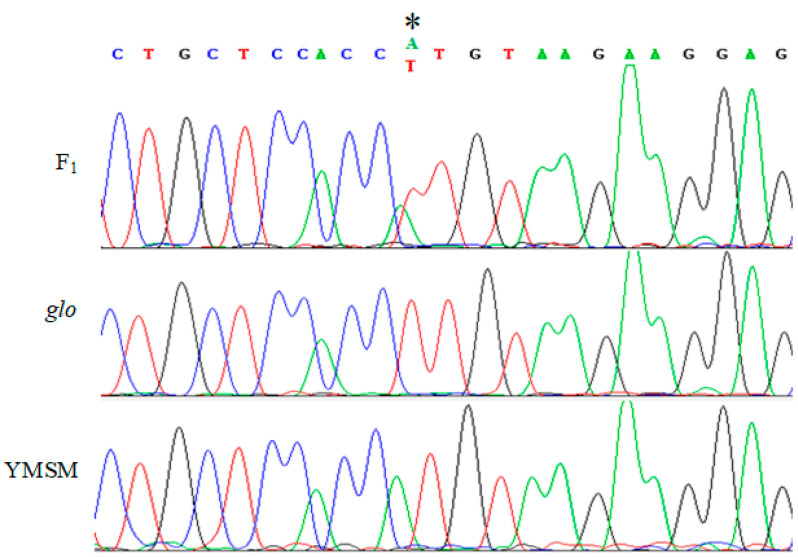
The sequencing results of SNP (Ch1:1522703) in YMSM YMSM, *ogl*, and F_1_. * indicates SNP (Ch1: 1522703).

The sequencing chromatogram of F_1_ plants showed that the amplification efficiencies of the two alleles were similar and there was no obvious competitive inhibition (Figure 3). To further verify the candidate gene, another 60 open-glume plants were randomly selected to extract DNA for PCR and every five PCR products were mixed and sequenced. The results showed that all open-glume plants contained homozygous *OsJAG^ogl^*. We amplified the entire genomic region of *OsJAG* from YMSM and *ogl* for sequencing. Referring to YMSM and R498, we did not find any new SNP in *OsJAG^ogl^*, except for SNP(Chr1:1522703) (Table 3).

### 2.4. Analysis of Splicing and Expression Patterns of OsJAG

**Figure 4:**
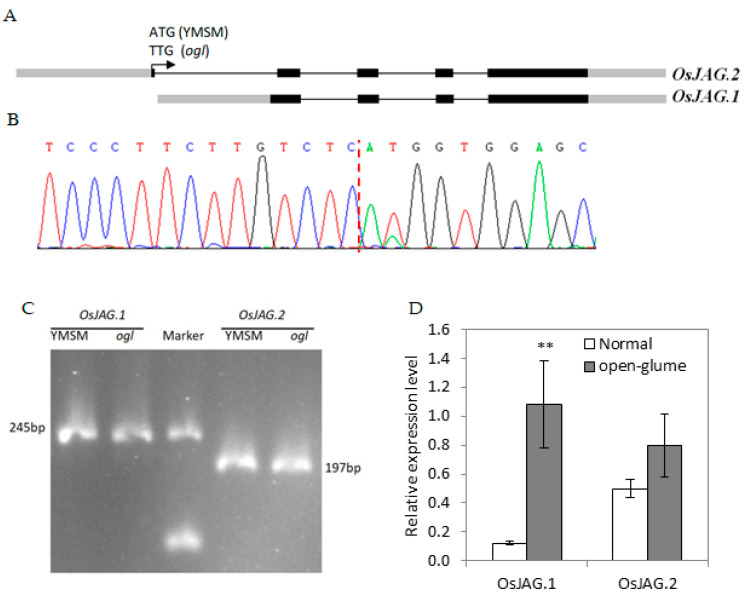
Analysis of the two expressed *OsJAG* transcripts. (**A**) Schematic diagram of the two transcripts of *OsJAG* denoted in NCBI. The grey box represents the untranslated sequence, the black box represents the coding sequence, and the black line represents the intron regions. The arrow indicated the start codon of *OsJAG.1*. (**B**) The reverse sequencing result of transcript *OsJAG*.2 in the F_1_ generation. The red dotted line indicates the splice site. (**C**) Agarose gel electrophoretic analysis of partial fragments of two transcripts of *OsJAG*. (**D**) The expression analysis of two transcripts in normal plants and open-glume plants from the F_3_ population. Values represent the mean ± SD of three biological replicates, with every replicate containing three young panicles from different plants. Student’s t-tests were used to generate *p*-values. (** *p* < 0.01).

According to NCBI, *OsJAG* produces two transcript variants XM_026023842.1 (*OsJAG*.2) and XM_015791728.2 (*OsJAG*.1) (Figure 4A). Compared to *OsJAG*.2, there are 12 additional nucleotides following the start codon in *OsJAG*.1. We amplified the fragments of *OsJAG*.2 from F_1_ cDNA. The sequence results supported the transcriptional prediction of *OsJAG*.2 from NCBI (Figure 4B). At the same time, the double peak in the sequencing chromatogram confirmed that the transcript *OsJAG.2^ogl^* was also present in the F_1_ plant. We also amplified the 5′ fragments of *OsJAG.1* and *OsJAG.2* with the cDNA from *ogl* and YMSM, respectively, and confirmed that both transcripts existed in *ogl* and YMSM (Figure 4C). We also compared the expression levels of *OsJAG*.1 and *OsJAG*.2 between normal plants and glume-open plants from the F_3_ population. We found that the expression level of *OsJAG*.1 was extremely significantly upregulated in glume-open plants (Figure 4D).

### 2.5. Expression Analysis of Genes Related to Floral Organ Development

**Figure 5:**
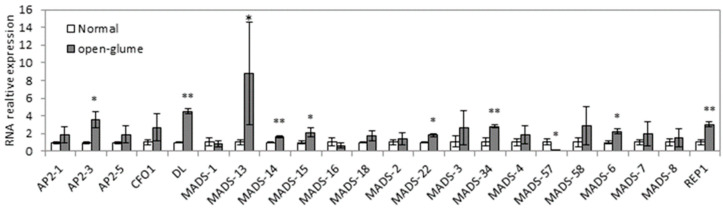
Expression analysis of genes related to floral development by quantitative RT-PCR. Values represent the means ± SD of three biological replicates. Student’s *t*-tests were used to generate *p*-values. (* *p* < 0.05, ** *p* < 0.01).

*OsJAG* is a key regulatory gene in the development of floral organs, indicating that there are many downstream genes regulated by it [29]. To further understand the function of *OsJAG*, we analyzed the expression of A/B/C/D/E-class genes in young panicles (5 mm in length) of normal plants and open-glume plants, respectively. The results showed that the expression of many genes was altered in the *ogl* mutant background (Figure 5), including upregulation of genes from class A (*OsMADS14/15)*, class C (*DL*), class D (*OsMADS13* and *REP1*), and class E (*OsMADS6/22/34*). Class B genes (*OsMADS2/16*) did not vary but some E-class genes (*OsMADS57*) were downregulated. *Ap2* family genes were previously demonstrated to regulate the development of rice florets [14] and our results demonstrated the upregulation of *AP2-3* in *ogl*. All of these results indicated that *OsJAG* could regulate the expression of A/B/C/D/E-class genes and *AP2* genes.

## 3. Discussion

A natural open-glume mutant, *ogl*, was originally discovered in the paddy field. The main characteristics of the *ogl* floret were that the lemma and palea could not clasp to each other, the number of stamens decreased, and the number of pistils increased (Figure 1). Genetic analysis and gene mapping were thus carried out (Table 1 and Figure 2) and a novel allele, referred to as *OsJAG^ogl^*, was identified (Figure 3). All of the randomly selected open-glume plants contained homozygous *OsJAG^ogl^*, indicating that *OsJAG^ogl^* resulted in the open-glume phenotype. The sequencing results of cDNA fragments confirmed that *OsJAG.2^ogl^* lost its original start codon. The functions of *OsJAG.1* and *OsJAG.2* are believed to be partially similar, so the upregulation of *OsJAG.1* in open-glume plants may be the result of self-regulation to compensate for the loss of *OsJAG.2^ogl^* function (Figure 4D).

In Arabidopsis thaliana, an ABCDE model of flower development has been established for decades [32,33,34] and has since been extended to flower development in monocotyledonous plants [35,36,37]. We compared the expression of ABCDE genes between normal plants and open-glume plants (Figure 5). Generally, C-class genes and D-class genes promote the development of carpels, so the upregulation of C-class genes and D-class genes might cause the transformation of stamens into pistils. E-class genes were both upregulated and downregulated in *ogl* mutants, suggesting that there are unknown factors involved in floret development in rice. Overexpression of OsMADS22 resulted in aberrant floral morphogenesis [38]. We detected the upregulated expression of OsMADS22 in o*gl*, suggesting that *OsMADS22* may mediate the phenotype of abnormal florets. A-class genes specify the identity of sepals [34]. Some A-class genes were upregulated in *ogl*, which might cause imbalanced growth of lemma and paleae and result in the open-glume phenotype. Complete inactivation of *OsJAG* leads to infertility [29]. In *ogl*, *OsJAG* partially lost its function as only one transcript of *OsJAG^ogl^* was inactivated (Figure 3 and Figure 4 and Table 3). The generation of a small number of homozygous seeds also supports the above inference (Figure 1G). The amino acid sequence determines protein function, and the amino acid sequence of *OsJAG1* is similar to that of *OsJAG2*, but the N-terminus of *OsJAG1* has four more amino acid residues than *OsJAG2*, suggesting that their functions are similar. However, our results show that *OsJAG1* can not completely replace the function of *OsJAG2* (Figure 1). The *sl1* florets displayed homeotic conversions and some stamens converted into stigmas [29]. The *ogl* florets also displayed a similar conversion, which indicated that *OsJAG.2* was also necessary for the development of stamens. The mutation *OsMADS16*(*spw1*) caused the transformation of stamens to carpels [39] and the expression of *OsMADS16* was downregulated in *sl1* [29], suggesting that *OsJAG* might regulate *OsMADS16* expression. However, no significant downregulation of *OsMADS16* was detected in *ogl* (Figure 5). Taken together, these results indicated that the functions of two transcripts of *OsJAG* need to be respectively studied and the mutant *ogl* will serve as a useful tool for this task in the future.

*OsJAG* is mainly expressed in the panicle [28,29,40] and the mutants ogl and sl1 showed no obvious phenotype during vegetative growth [29], which suggested that *OsJAG* mainly affected floral organ development. Interestingly, the homologous gene of *OsJAG* in *Arabidopsis thaliana* did not show similar homeotic conversions during flower development [30,41,42]. The transformation from stamen to stigma was not observed even in the double mutant *jag nub* (*AtNUB* has produced possible functional redundancy of *AtJAG* in *Arabidopsis*) [43], suggesting that *OsJAG* may have evolved its homeotic conversions function after the lineage split between monocotyledons and dicotyledonous plants. Overexpression of *OsJAG*.1 led to a decrease in plant height [29], suggesting that *OsJAG*.1 has a potential function in regulating vegetative growth, which is similar to that of *AtJAG* in Arabidopsis [30,43]. Weak expression of *OsJAG* was also detected in roots and leaves by semiquantitative RT–PCR and GUS staining [28]. It is suggested that *OsJAG* may regulate vegetative growth in an unknown way, even if the effect is not great.

The phenotypes of *ogl* were similar to those of OsMADS1 mutants, such as the abnormal glume and a decreased number of stamens (Figure 1A–I) [19,20,22]. The *OsMADS1–*microRNA172*–AP2* model controls floret development in rice [14]. We detected the expression of *OsMADS1* and three *AP2* genes and *AP2-3* was significantly upregulated in *ogl*, suggesting that the phenotype of *ogl* is not seemingly related to the *OsMADS1–microRNA172–AP2* pathway, although *OsJAG* can affect the expression of some *AP2* genes.

## 4. Materials and Methods

### 4.1. Plant Materials

YMSM (Yuanmeisimiao) is an *indica* rice cultivar developed by crossbreeding, which displays normal floret development. The *ogl* mutant was originally discovered in the late season of 2020. Since the surrounding cultivars were *indica* rice, we deduced that *ogl* is of *indica* background. Since its original wild type is unknown, YMSM was used as a control in the phenotype analysis. All materials are cultivated in the normal season at the Baiyun Experimental Station of Guangzhou, Guangdong Province, China.

### 4.2. BSA Sequencing and SNP Analysis

Segregating F_2_ populations were derived from the cross of *ogl* (female parent) with YMSM (male parent). After heading, 20 plants with glume opening florets and 20 plants with normal florets were randomly selected from the segregating population. The genomic DNA of each plant was extracted from the leaves. The quality and concentration of the extracted DNA were tested by the nanodrop spectrophotometer. An equal amount of DNA from plants exhibiting similar glume phenotypes was mixed to construct the extreme phenotype mixing pool. Two extreme pools, normal floret and open glume floret, were sent to Higentec Technologies Co., Ltd. (Hunan, China) for genome sequence and SNP analysis with the genome of R498 as the reference (www.mbkbase.org/R498/, accessed on 10 January 2023). SNPs with a read support of less than 4 were filtered out.

### 4.3. SNP Analysis

Based on the upstream and downstream sequence information of SNP, primers were designed by online software (www.ncbi.nlm.nih.gov/tools/primer-blast/, accessed on 20 March 2023). The fragments containing the SNP were PCR-amplified with KOD-FX(TOYOBO) and sequenced by Sangon Biotech (Shanghai) to check the SNP genotype.

### 4.4. Microscopic Observations of Glume Sections

Floret samples from the *ogl* and YMSM plants were collected the day before heading and fixed in FAA solution. Dehydration and infiltration steps were carried out and then embedded in paraffin. Tissue cross sections (10 μm in thickness) were cut into semithin sections and fished out onto the microscope slides. The slides were kept in a 1% (*w*/*v*) Toluidine blue solution for 2 min, washed with running water, differentiated with 95% alcohol, and observed under a Nikon optical microscope (Nikon Eclipse E100, Tokyo, Japan).

### 4.5. Gene Expression Analysis

The open-glume plants and normal plants were selected based on our SNP analysis (Chr1: 1522703) from the F_3_ population and 10 panicles were collected from different individual plants displaying these phenotypes and stored in liquid nitrogen when the panicles were 5 mm in length. The total RNA was extracted using Trizol (Invitrogen, Waltham, MA, USA) according to the manufacturer’s instructions and was treated with DNase I (Takara, Beijing, China) to remove genomic DNA contamination. The quality and concentration of the extracted RNA were checked using the nanodrop spectrophotometer. First-strand cDNAs were synthesized from 1 μg total RNA using the EvoM-MLV First-Strand cDNA Synthesis Kit (Accurate Biology, Changsha, China). qRT-PCR was performed using SYBR Green Mix (Accurate Biology, Changsha, China) on a CFX Connect™ Real-Time PCR Detection System (Bio-Rad, Hercules, CA, USA). The *OsEF1α* gene was used as an endogenous control. The primers used for qRT-PCR are listed in Supplementary Materials Table S1.

### 4.6. I_2_-KI Staining of Pollen Grains

Anthers were sampled 2–3 h before heading, placed on the slide, mashed, and stained with a 1% iodine potassium iodide (I_2_-KI) solution. The staining pattern was observed using a BK5000-FL (Optec, Shenzhen, China) microscope. One anther was selected from each floret and 10 anthers each from YMSM, and *ogl* were sampled for I_2_-KI staining analysis.

## 5. Conclusions

In this paper, a rice mutant, *ogl*, was reported and its phenotypic characteristics were described in detail. Genetic analysis and bulked segregation analysis showed that the open-glume phenotype was controlled by one recessive gene and *OsJAG^ogl^* was identified as the candidate gene. *OsJAG* encodes two versions of transcripts. Compared to *OsJAG.*2, there are 12 additional nucleotides following the start codon in *OsJAG*.1. In open-glume plants, the start codon of *OsJAG*.2 is mutated and the expression of *OsJAG*.1 is upregulated, which suggests that *OsJAG*.2 plays an indispensable role in flower development. The expression level of multiple flower development-related genes was altered in open-glume plants and the expression trends of associated genes were different from that in *OsJAG* (*SL*1) [29], which indicates that the functions of the two transcripts of *OsJAG* need to be further studied. Thus, this study provides new genetic material and preliminary results for further study of the function of *OsJAG* in rice-floret development.

## Figures and Tables

**Table 1:** Segregation analysis of the F_2_ population.

Family Line	F_2_				
	No. of Plants with Normal Phenotype	No. of Plants with Open-Glume Phenotype	Ratio (Normal/Open-Glume)	Total Plants	χ^2^ (3:1)
1	381	63	6.05	444	27.10
2	251	32	7.84	283	27.57
3	188	17	11.06	205	29.63

χ^2^_(0.05, 1)_ = 3.84 (3∶1).

**Table 2:** Seven SNPs in candidate regions.

Position	Ref	Alt	Anno	Gene ID	Function	High Expression Tissue	
Chr1:626540	A	T	stop-loss	OsR498G0100035900.01	Conserved hypothetical protein	Root	
Chr1:667077	G	T	stop-gain	OsR498G0100039300.01	Uncharacterized protein	Root tip and endosperm	
Chr1:704735	G	A	stop-gain	OsR498G0100042300.01	Nucleoside triphosphate hydrolase	Endosperm	
Chr1:1389031	A	T	stop-gain	OsR498G0100088600.01	3-hydroxyisobutyryl-CoA hydrolase-like protein 5	Leaf and root	
Chr1:1522703	A	T	start-loss	OsR498G0100098300.01	Zinc finger protein	Panicle and callus	
Chr1:3175821	G	T	stop-gain	OsR498G0100212200.01	Receptor-like protein	Pollen	
Chr1:3188476	G	T	stop-gain	OsR498G0100212200.01	Receptor-like protein	Pollen	

**Table 3:** Haplotypes derived from R498 YMSM and *ogl*.

Position	1522703	1523044	1523295	1523590	1523663	1523768	1524171	1524265
R498	A	T	G	A	A	G	A	A
YMSM	A	C	G	A	G	G	C	G
*ogl*	T	C	A	T	A	T	C	G
Annotation	Startloss	Intron	Ser-Asn	Intron	Synonymous	Intron	Intron	Synonymous

## Data Availability

The data presented in this study are available upon request from the corresponding authors.

## Supplementary Materials

The following supporting information can be downloaded at: https://www.mdpi.com/article/10.3390/ijms241612702/s1. Table S1, List of primers used in this study.
