# Supplementary material for: Characterization and Gene Mapping of an Open-Glume Oryza sativa L. Mutant
Source: Int J Mol Sci. 2023 Aug 11;24(16):12702. doi: 10.3390/ijms241612702 (PMC10454609; doi:10.3390/ijms241612702)
Supplement: Supplementary file 1 [file ijms-24-12702-s001.zip › ijms-2538065-supplementary.pdf]

Table S1 List of primers used in this study

Primer name	Sequence（5'-3'）	Purpose
K3F	CGCGTCTCAAGTCGATCTCA	SNP analysis
K3R	CTGTGCCGACAACAAGAATGAA	
JAG.1reF	CTCATCATTGTGCAAACAGGAAC	Analysis of two transcripts
JAG.1reR	CTTCTTCTTAGTAACCCTGGATGCT	
JAG.2reF	CTCTCCTTCCAAAAGCCTCATT	
JAG.2reR	CTTCTTCTTAGTAACCCTGGATGCT	
EF1α-reF	TTTCACTCTTGGTGTGAAGCAGAT	Expression analysis
EF1α-reR	GACTTCCTTCACGATTTCATCGTAA	
AP2-1reF	GCCCAACCTCATCCCCTATT	
AP2-1reR	CTGCTTCCATGCCCAGCT	
AP2-3reF	ACAGGGGCGTCACCTTCTACA	
AP2-3reR	GCACAAACTCCTCCTTGGTCC	
AP2-5reF	ATTACGAGGATGACCTGAAGCA	
AP2-5reR	CGAACAGCCCCAAGTAGACG	
CFO1reF	CTGAAGGATGTGGAATGTGG	
CFO1reR	AACACTGGCTCTAAGCAGCA	
DLreF	CCCATCTGCTTACAACCGCTT	
DLreR	GTTGGAGGTGGAAACCGTCG	
MADS13reF	ATGGGGAGGGGCAGGATTGAG	
MADS13reR	TGCGCCTTCTTGTACCTGTCA	
MADS14reF	CGGTTGCGAGACGAGGAA	
MADS14reR	GAAAGACGGTGCTGGACGAA	
MADS15reF	CGTCGTCGGCCAAACAG	
MADS15reR	TGACTTCAATTCATTCAAGGTTGCT	
MADS16reF	CGAGGCGTACGAGACTCTGC	
MADS16reR	ACCACGCGGAAGGCGAACAT	
MADS18reF	AGCCAAATACTGAGGACC	
MADS18reR	TTGCTGGAGTTCTTTTATTGT	
MADS1reF	CCCAGATCAGGGTGACCATT	
MADS1reR	GGGTGATGAGCAACCATGTC	

MADS22reF	TATCATCCTTGTGGCTGCAT
MADS22reR	TGGGATAATTAAACGGCACA
MADS2reF	CCACCACTGGATTGAATGTCC
MADS2reR	CAGAATGGCAAGCACAGAGC
MADS34qF	CAACCAGAGCACTTCTTCCA
MADS34qR	CTGAAGCTGAAACGGTAGCT
MADS3reF	AACGCAAACAGTAGGACCATAGTG
MADS3reR	CCCCTCTCATTCTCAACAACC
MADS4reF	AGCACAAGATGTTGGCTTTTAGGG
MADS4reR	CATCTAGCAGCGCATGAGG
MADS57reF	CCGTACAAGCTGGACGATAA
MADS57reR	ACATGAGCATCCGACAACAT
MADS58reF	GAGCAAAGTTGCTGAGAGTG
MADS58reR	GAGGCTGATGCATGATGTTG
MADS6reF	GGAAGAGTTGAGCTGAAGCG
MADS6reR	AGAGAACGGACAGCTCGTAG
MADS7reF	TGGGTTCTTCCATCCACTTG
MADS7reR	CGTCATCATCATGGTAGCCA
MADS8reF	CACCTTGCAGATCGGGTTTA
MADS8reR	ATCTGTGTCGTCACATCCGT
REP1reF	GAAAGGAGACGCGGACAAG
REP1reR	CCGGTGTTGTTGCTCATCAT
